# Survival status and predictors of mortality among preterm neonates admitted to the neonatal intensive care unit at public hospitals of Harari region and Dire Dawa administration, eastern Ethiopia: Retrospective cohort study 2025

**DOI:** 10.1371/journal.pone.0347244

**Published:** 2026-04-17

**Authors:** Boru Abera Ebsa, Maleda Tefera, Dawit Tamiru, Abraham Negash, Naol Oda, Merga Dheresa

**Affiliations:** 1 School of Midwifery, College of Health and Medical Sciences, Haramaya University, Harar, Ethiopia; 2 School of Nursing, College of Health and Medical Sciences, Haramaya University, Harar, Ethiopia; Federal University of Sergipe, BRAZIL

## Abstract

**Background:**

The neonatal period is the most vulnerable time for an infant’s survival, particularly for preterm neonates. Preterm birth is among the leading causes of neonatal mortality. Many neonatal complications can be prevented, but preterm birth remains a leading cause of admission, death, and long-term complications, highlighting the need for further research on outcome and survival disparities across populations and settings. Therefore, this study aimed to assess survival status and predictors of mortality among preterm neonates admitted to neonatal intensive care units at public hospitals in the Harari region and Dire Dawa administration, Eastern Ethiopia, from November 1, 2021 to October 30, 2024.

**Methods:**

The hospital-based retrospective cohort study was conducted among preterm neonates admitted to the neonatal intensive care unit at public hospitals of the Harari region and Dire Dawa administration, Eastern Ethiopia. A simple random sampling technique was used, and data were extracted from neonates’ medical records and registration formats using a structured checklist prepared in English. Descriptive statistics, life table, Kaplan-Meier curves, and Log-rank test were used to estimate and compare survival time. Predictors of mortality were identified using the Cox Proportional Hazard model.

**Results:**

Out of 612 preterm neonates, 205 (33.5%; 95% CI: 29.76–37.39) died, corresponding to an incidence rate of 52.76 deaths per 1,000 preterm neonate-days (95% CI: 46.01–60.50), with a median survival time of 18 days. As multivariable cox-regression result,  ≥ 4 antenatal care contact (AHR = 0.56; 95% CI: 0.36–0.89), receiving KMC (AHR = 0.16; 95% CI: 0.09–0.27), 5th minute APGAR score <7 (AHR = 1.80; 95% CI: 1.22–2.66), PNA (AHR = 1.55; 95 CI: 1.08–2.22), resuscitation with bag and mask at birth (AHR = 1.59; 95% CI: 1.10–2.29), RDS (AHR = 1.75; 95% CI: 1.22–2.51), born in non-cephalic presentation (AHR = 1.68; 95% CI: 1.12–2.53), and neonatal sepsis (AHR = 1.58; 95% CI: 1.09–2.28) were identified as significant predictors of preterm neonates mortality.

**Conclusion:**

The incidence of preterm neonatal mortality was high in this study. Adequate Antenatal care (ANC) and kangaroo mother care (KMC) significantly improved preterm survival, while low APGAR score, resuscitation with bag and mask, neonatal sepsis, PNA, and RDS were major predictors of preterm neonatal death. Emphasis should be placed on strengthening antenatal and perinatal care, along with early detection and management of identified neonatal complications.

## Introduction

Preterm birth is defined as a live birth born before 37 completed weeks of pregnancy or less than 259 days from the first date of the woman’s last normal menstrual period [1,2]. Preterm birth is classified as very preterm (28 to < 32 weeks), moderate preterm (32 to <34 weeks), and late preterm (34 to <37 completed weeks) based on gestational age [1,3]. The majority of preterm births occur spontaneously, while others are caused by medical conditions like infections or other pregnancy complications that require early labor induction or cesarean delivery [1].

Globally, the preterm birth rate was reduced slightly from 10.6% in 2014 to 9.9% in 2020, with 15% of all preterm births occurring at <32 weeks of gestation [2,4]. Despite this reduction, the burden is disproportionately high, as over 50% of all preterm births occurred in eight countries. Highest in India, followed by Pakistan, Nigeria, China, Ethiopia, Bangladesh, the Democratic Republic of the Congo, and the USA [4]. Southern Asia and sub-Saharan Africa contributed to approximately 65% of all preterm births globally in 2020 [4]. The majority of preterm births and related mortality occur in low- and middle-income countries, and their burden is high in sub-Saharan Africa and South Asia [5].

Preterm birth is a major global health challenge, significantly contributing to neonatal and under-five mortality. Preterm birth affects an estimated 13.4 million newborns annually, accounting for 9.9% of all births [1,4] and it is the leading cause of death in children under five, responsible for 1 million deaths, or 35% of neonatal deaths and 18% of under-five deaths worldwide [1,5].

Preterm birth remains a health problem both in developed and developing countries, with notable disparities. In developed settings, such as the USA, the preterm mortality rate is 22.35 per 1000 births [6]. In middle-income countries like China, regional disparities are observed, with preterm-related neonatal deaths accounting for 51.3% in eastern and 44.5% in western regions [7]. Developing countries carry the highest burden of preterm mortality. India alone accounts for nearly one-third (33%) of global preterm deaths [5].

Sub-Saharan Africa bears the highest burden of neonatal mortality, as the 2022 World Health Organization (WHO) report identified the region as having neonatal mortality of 27 per 1000 live births [8]. Evidence from the Alliance for Maternal and Newborn Health Improvement (AMANHI) study further highlights that preterm birth accounts for 46% of neonatal mortality, of which 40% occurs in sub-Saharan Africa [9]. Country-level evidence reported the severity of the problem, with preterm birth complications responsible for 36% of neonatal deaths in South Africa and ranking as the second major cause in Kenya (28%) [10].

In East Africa, preterm neonatal mortality remains unacceptably high, with Uganda reporting a rate of 31.6% [11]. Ethiopia ranks among the top five globally for both preterm births and neonatal deaths, with a preterm birth prevalence of 10.48% [4,12,13]. The country’s national neonatal mortality rate (33 per 1000 live births) in 2019 exceeded the regional averages of Sub-Saharan Africa and Central and Southern Asia [8,14,15]. Regional variations within Ethiopia also demonstrate a preterm neonatal mortality rate ranging from 22.7% to 36.1% [16,17]. Specifically, in eastern Ethiopia, preterm complications contribute to 28.8% of neonatal deaths [18].

Beyond survival, preterm birth has lasting health, social, and economic consequences. Affected newborns face neurodevelopmental delays, sensory and cognitive impairments, and behavioral challenges [19,20]. Mothers experience disrupted early bonding, postpartum health issues, and reduced emotional well-being [19]. Economically, preterm births impose heavy costs on families, including medical expenses and caregiving demands, special education needs, lost work time, and reduced productivity [21].

Ethiopia’s current neonatal mortality rate remains unacceptably high, falling short of the Health Sector Transformation Plan-II (HSTP-II) target of 21 per 1,000 live births by 2024/25 and raising uncertainty about achieving the Sustainable Development Goal target (SDG) of 12 per 1,000 live births by 2030 [8,15,22]. Despite national strategies and interventions, preterm-related mortality remains persistently high. However, evidence on the survival status and predictors of mortality among preterm neonates remains limited, particularly in the current study area. Most existing studies have been conducted in the northern, southwestern, and central regions, leaving a significant geographic gap. Additionally, findings from previous studies are often inconsistent, with neonatal outcomes varying across regions, facilities, and time periods. To address these gaps, the present study uses a larger sample size than previous research and aims to provide context-specific evidence on preterm neonatal survival. Specifically, it seeks to assess the survival status and predictors of mortality among preterm neonates admitted to Neonatal Intensive Care Units (NICUs) at public hospitals in the Harar region and Dire Dawa Administration, Eastern Ethiopia.

## Methods and materials

### Study setting

The study was conducted at public hospitals in the Harar region and the Dire Dawa Administration, Eastern Ethiopia. The Harari region is one of Ethiopia’s regional states, with an estimated population of 283,000, comprising 143,000 males and 140,000 females [23]. The capital city of the Harari region is Harar, an ancient city situated on a hilltop in eastern Ethiopia, approximately 526 kilometers from Addis Ababa. The region has two public hospitals: Hiwot Fana Comprehensive Specialized University Hospital (HFCSUH) and Jugal General Hospital (JGH). The Dire Dawa administration is located 515 kilometers northeast of Addis Ababa, the capital of Ethiopia. It is one of the two chartered cities in the country, with an estimated population of 551,000, including 278,000 males and 273,000 females [23]. The two public hospitals in Dire Dawa are Dilchora Referral Hospital and Sabian General Hospital.

### Study period

This study was conducted among preterm neonates admitted to the NICU of public hospitals in the Harari region and Dire Dawa administration from November 1, 2021 to October 31, 2024, and the preterm neonates’ medical charts and registers were reviewed from January 1–31, 2025.

### Study design

The hospital-based retrospective cohort study was conducted.

### Population

#### Source population.

All preterm neonates admitted to the NICU at public hospitals in the Harar region and the Dire Dawa administration.

#### Study population.

All randomly selected preterm neonates who were admitted to the NICU at public hospitals in the Harar region and the Dire Dawa administration from November 1, 2021, to October 31, 2024. A preterm neonate’s medical card with incomplete records of date of admission, date of discharge, gestational age, and unknown status was excluded.

### Sample size and sampling procedure

The sample size was calculated using the STATA statistical package (version 17.0) for Cox PH regression, considering assumptions such as a hazard ratio (1.54) for selected covariates (having respiratory distress syndrome (RDS) and a failure (death) probability of 29.7%, based on a previous study conducted at Tikur Anbessa Specialized Hospital in Addis Ababa, Ethiopia. [24]. Assuming a type I error (α) of 0.05, a study power of 80%, and a 10% rate of withdrawals or incomplete records. RDS was selected among five covariates because it yielded the largest sample size. Consequently, the calculated maximum sample size for this study was 630. The medical cards of preterm neonates were identified using their Medical Record Number (MRN) through the NICU registration as the sampling frame. Then, a simple random sampling method was applied, and the number of medical charts was generated using Excel to proportionally select 630 preterm neonate charts from each hospital for the study.

### Variables of the study

Survival status of preterm neonates is the study’s dependent variable. Socio-demographic characteristics, obstetrics and gynecological related factors, maternal medical conditions, and preterm neonate-related characteristics are all independent variables.

### Operational definitions

#### Survival status:

the outcome of preterm neonates, either death or censored.

#### Censored:

preterm neonates those alive beyond 28 days after birth, discharged with parental refusal, discharged with improvement, and referred to other health institutions.

#### Follow-up time:

starting from the date of admission up to 28 days of life.

#### Time to death:

is the time in days from admission to NICU until death/transfer/discharge of neonates in the first 28 days of life.

#### Event

: the death of preterm neonates after admission to the NICU.

#### Time scale:

the survival time measured in days.

#### Preterm neonatal sepsis

: defined as clinical signs and symptoms with the presence of risk factors, lab tests, or confirmed by blood culture or diagnosed by a physician as the presence of sepsis [25].

**Bad obstetric history:** implies previous unfavorable fetal outcome in terms of two or more consecutive spontaneous abortions, early neonatal deaths, stillbirths, intrauterine fetal deaths, and intrauterine growth retardation [26].

### Data collection instrument

The data collection checklist includes information on socio-demographic characteristics, obstetrics and gynecological related factors, maternal medical conditions, preterm neonatal status, and characteristics related to preterm neonates. Research assistants were trained for two days so that they became familiar with the objectives of the study, its contents, sampling procedure, data extraction tools, and the issue of confidentiality. The data extraction checklist was adapted by reviewing various literature from similar studies and modified using preterm neonates’ medical records and NICU health management information system (HMIS) registration format [24,25,27–30]. Two bachelor’s degree-holder Midwives, data collectors, were used as data extractors from the neonate’s medical card and logbook, and supervised by an MSc holder Midwife. During the data extractions, regular supportive supervision and discussions with data collectors and the supervisor were conducted. On-site checking and review of the completed checklist were done by the responsible body.

### Data management and quality

Before actual data collection, the records were reviewed, and preterm neonatal cards were identified by medical record number. Then, the data were extracted from each preterm neonate’s medical records and health management information system (HMIS) registration format, using a structured checklist prepared in English from those preterm neonates’ medical records that fulfilled the inclusion criteria. A pretest was conducted on 5% of the total sample size using neonates’ medical records and register books at Hiwot Fana Comprehensive Specialized Hospital during a period outside the main study. Following the pre-test, checklist modifications were made to improve the instrument’s clarity, consistency, and reliability.

### Methods of data processing and analysis

The data were coded and exported into STATA version 17.0 for analysis. The Kaplan–Meier survival curve was used to estimate the median survival time and cumulative survival probability, and to compare survival experiences across different covariates. The log-rank test was applied to statistically assess differences in survival across categories of the explanatory variables. A life table was employed to estimate the cumulative survival probability at different time intervals.

A bivariable analysis using crude hazard ratios was performed to identify candidate variables (p < 0.25) for inclusion in the multivariable Cox regression to identify predictors of preterm neonatal death. The proportional hazards assumption was evaluated using the Schoenfeld residuals test (global test), and the overall model fitness was checked graphically by using the Cox-Snell residual plot.

The Cox proportional hazards model was used to identify significant predictors of mortality and time to death among preterm neonates. The Adjusted Hazard Ratios (AHR) with 95% Confidence Intervals (CI) were used to evaluate the association between predictors and preterm neonatal death. Statistical significance was reported at p-value < 0.05.

### Ethical considerations

Ethical approval was obtained from the Institutional Health Research Ethical Review Committee (IHRERC) **(Ref. No. IHRERC/227/2024)** of Haramaya University, College of Health and Medical Sciences. After approval, an official letter was sent from Haramaya University, College of Health and Medical Sciences, to the director of HFCSUH, Jugal General Hospital, and the Dire Dawa Administration health bureau. Informed, voluntary, written, and signed consent was secured from each hospital’s head. The confidentiality of neonates was preserved through anonymous data collection.

## Results

### Socio-demographic characteristics

In the final analysis, 612 (97.14%) medical records of preterm neonates meeting the inclusion criteria were reviewed, whereas 18 (2.86%) records were excluded due to incomplete data. Out of incomplete cards, 6 had no date of admission, 7 had no date of discharge, and 5 were not available during card review. The average maternal age was 26.90 years with a standard deviation of ±5.50 years. More than two-thirds (488; 79.74%) of the maternal ages were between 20 and 34 years. Slightly more than half (315; 51.47%) of preterm neonates were male (Table 1).

**Table 1:** Socio-demographic characteristics of mothers of preterm neonates admitted to the NICU of public hospitals in the Harari region and Dire Dawa administration from November 1, 2021, to October 31, 2024 (n = 612).

Characteristics		Frequency	Percentage (%)
Hospital	HFCSUH	379	61.93
	Jugal General Hospital	58	9.48
	Dilchora Referral Hospital	96	15.69
	Sabian General Hospital	79	12.91
Maternal Age	15 – < 20 years	51	8.33
	20-34 years	488	79.74
	≥ 35 years	74	11.93
Maternal residence	Urban	273	44.61
	Rural	339	55.39
Sex of neonates	Male	315	51.47
	Female	297	48.53

### Obstetrics and medical-related factors

Among the 612 mothers of preterm neonates, 474 (77.45%) had antenatal care follow-up, of whom 299 (48.86%) had fewer than four ANC contacts. Nearly half of the neonates were born to multiparous mothers 274 (44.77%), and labor started spontaneously in 427 (69.77%) cases. Most neonates were from singleton pregnancies 506 (82.68%) and were delivered at hospitals (88.56%). Spontaneous vaginal delivery accounted for 437 (71.41%) births, with a median labor duration of 6 hours. Preeclampsia was present in 140 (22.88%) mothers, preterm premature rupture of membranes occurred in 217 (35.46%), and 179 (29.25%) mothers received antenatal corticosteroids. Additionally, anemia was observed in 61 (9.97%) mothers (Table 2).

**Table 2:** Obstetrics and Gynecological Related Factors of mothers among preterm neonates admitted to the NICU of public hospitals in the Harari region and Dire Dawa administration from November 1, 2021 to October 31, 2024 (n = 612).

Characteristics		Frequency	Percentage
ANC follow-up contacts	No ANC follow-up	138	22.55
	1-3	299	48.86
	≥4	175	28.59
Gravidity	Primigravida	191	31.21
	Multigravida (2–4)	280	45.75
	Grand multigravida (≥ 5)	141	23.04
Parity	Primipara	212	34.64
	Multipara (2–4)	274	44.77
	Grand multipara (≥ 5)	126	20.59
Bad obstetrics history	Yes	62	10.13
	No	550	89.87
Type of pregnancy	Singleton	506	82.68
	Multiple	106	17.32
Onset of labor	Spontaneous	427	69.77
	Elective/emergency Cesarean section	112	18.30
	Induced	73	11.93
Place of birth	Hospital	542	88.56
	Health center	59	9.64
	Home delivery	11	1.80
Mode of delivery	Spontaneous Vaginal Delivery	444	72.55
	Caesarean section	168	27.45
Duration of labor in hours	< 4	150	24.51
	4-18	408	66.67
	>18	54	8.82
Preeclampsia	Yes	140	22.88
	No	472	77.12
Eclampsia	Yes	41	6.70
	No	571	93.30
Antepartum hemorrhage (APH)	Yes	94	15.36
	No	518	84.64
PPROM	Yes	217	35.46
	No	395	64.54
Polyhydramnios	Yes	14	2.29
	No	598	97.71
Oligohydramnios	Yes	13	2.12
	No	599	97.88
Chorioamnionitis	Yes	6	0.98
	No	606	99.02
Antenatal steroid use	Yes	179	29.25
	No	433	70.75
Maternal Anemia	Yes	61	9.97
	No	551	90.03

PPROM: Preterm Premature Rupture of Membrane.

### Neonatal related characteristics

Among the 612 preterm neonates included in the study, the majority were late preterm (34 to <37 weeks, 46.08%), followed by moderate preterm (32 to <34 weeks, 29.90%) and very preterm (28 to <32 weeks, 24.02%). Most neonates were appropriate weight for gestational age (70.59%), with nearly two-thirds classified as low birth weight (64.54%). The majority had a 5-minute APGAR score ≥7 (73.04%), while nearly one-third required resuscitation at birth (30.23%). Common clinical conditions included respiratory distress syndrome (50.49%), hypothermia (76.47%), and neonatal sepsis (63.73%), with a smaller proportion developing perinatal asphyxia (15.69%). Most neonates had a cephalic presentation at birth (85.46%), and supportive interventions included kangaroo mother care (38.56%) and CPAP (57.52%) (Table 3).

**Table 3:** Neonatal-related characteristics among preterm neonates admitted to the NICU of public hospitals in the Harari region and Dire Dawa administration from November 1, 2021 to October 31, 2024 (n = 612).

Characteristics		Frequency	Percentage
Gestational age in weeks	28 to < 32 completed weeks	147	24.02
	32 to <34 completed weeks	183	29.90
	34 to <37 completed weeks	282	46.08
Weight of neonate (gm)	< 1000	20	3.27
	1000-1499	170	27.78
	1500-2499	395	64.54
	≥2500	27	4.41
Weight for gestational age at birth	Appropriate	432	70.59
	Small	180	29.41
APGAR score at 1^st^ minute	<7	305	49.84
	≥7	307	50.16
APGAR score at 5^th^ minute	<7	165	26.96
	≥7	447	73.04
Bag and mask resuscitation at birth	Yes	185	30.23
	No	427	69.77
Newborns temperature within 1 hour of admission	≤33^o^C	31	5.07
	33.1-35 °C	231	37.75
	35.1-36.4 °C	234	38.24
	≥36.5 °C	116	18.95
Newborn diagnosed with Perinatal asphyxia (PNA)	Yes	96	15.69
	No	516	84.31
Newborn diagnosed with respiratory distress syndrome	Yes	309	50.49
	No	303	49.51
Hypothermia diagnosed at admission	Yes	468	76.47
	No	144	23.53
Hypoglycemia diagnosed at admission	Yes	66	10.78
	No	546	89.22
Jaundice	Yes	112	18.30
	No	500	81.70
Newborn diagnosed with sepsis	Yes	390	63.73
	No	222	36.27
Congenital anomalies	Yes	22	3.53
	No	590	96.41
Newborn diagnosed with Anemia	Yes	18	2.94
	No	594	97.06
Type of presentation	Cephalic	523	85.46
	Non-cephalic	89	14.54
Neonates diagnosed with NEC	Yes	48	7.84
	No	564	92.16
The newborn received kangaroo mother care	Yes	236	38.56
	No	376	61.44
Neonate received continuous positive airway pressure (CPAP)	Yes	352	57.52
	No	260	42.48
Neonate received photo therapy	Yes	86	14.05
	No	526	85.95
Newborn heated with a radiant warmer	Yes	437	71.41
	No	175	28.59

### Major causes of preterm neonatal death in the NICU of public hospitals

**Fig 1:**
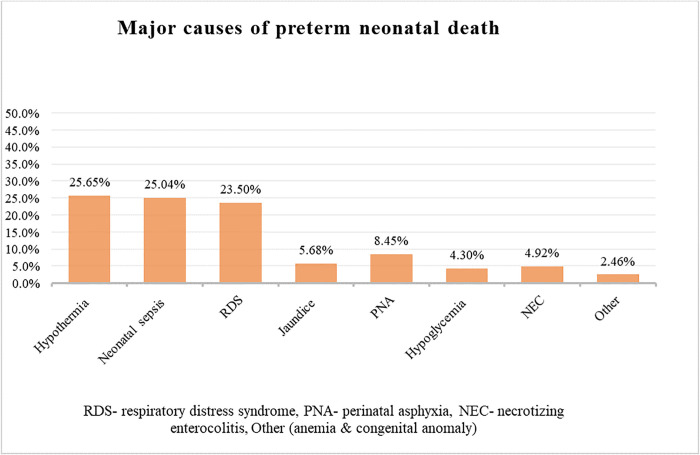
Distribution of causes of death among preterm neonates admitted to the NICU of public hospitals in the Harari region and Dire Dawa administration from November 1, 2021, to October 31, 2024.

The findings of this study revealed that multiple comorbidities were observed among deceased preterm neonates, significantly contributing to the overall burden of morbidity, resulting in preterm neonate mortality. The major causes of death among preterm neonates were hypothermia (25.65%), neonatal sepsis (25.04%), and respiratory distress syndrome (23.50%), together accounting for the majority of reported deaths (Fig 1).

### Survival status of preterm neonate

**Fig 2:**
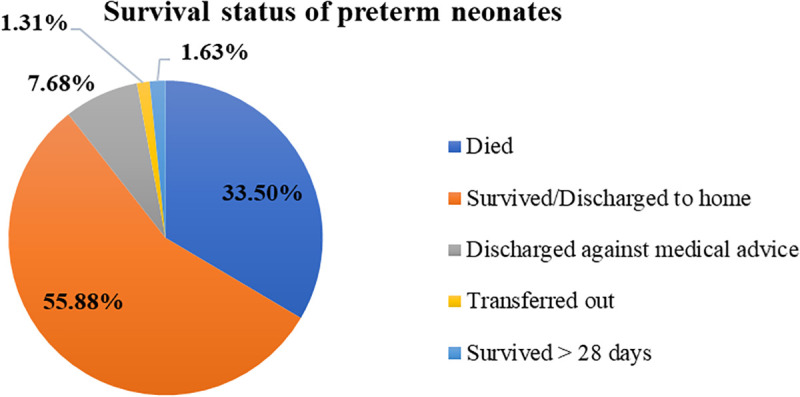
Overall survival status of preterm neonates admitted to the NICU of public hospitals in the Harari region and the Dire Dawa administration from November 1, 2021 to October 31, 2024.

Among admitted preterm neonates, 205 (33.50%; 95% CI: 29.76–37.39) died, 342 (55.88%) were discharged home alive, 47 (7.68%) had loss to follow up, 8 (1.31%) were referred to other health institutions, and 10 (1.63%) survived beyond 28 days (Fig 2).

The overall incidence rate of mortality was 52.76 deaths per 1,000 neonate-days (95% CI: 46.01–60.50), with a total follow-up time of 3,885.35 neonate-days among 612 preterm neonates. The median survival time was 18 days, while the median length of hospital stay was 4 days (IQR: 2–9 days).

The Kaplan–Meier survival analysis showed that 65 (31.71%) of all preterm neonatal deaths occurred on the first day of follow-up. The cumulative survival probabilities at 1, 4, and 28 days were 89.16% (95% CI: 86.39–91.40), 73.17% (95% CI: 69.22–76.70), and 44.38% (95% CI: 35.84–52.57), respectively (Table 4).

**Table 4:** Survival probability from life table among preterm neonates admitted to the NICU of public hospitals in the Harari region and Dire Dawa administration from November 1, 2021 to October 31, 2024 (n = 612).

Length of hospital stay (days)	Total at the beginning	Death	Censored	Survival probability (%)	95% Confidence interval
(0-4]	612	135	123	75.48	(71.66–78.86)
(4-8]	354	46	128	63.50	(58.94–67.70)
(8-12]	180	10	76	59.03	(53.99–63.72)
(12-16]	94	7	39	53.49	(47.41–59.18)
(16-20]	48	5	14	46.96	(39.36–54.19)
(20-24]	29	1	7	43.35	(34.86–51.53)
(24-28]	31	0	21	43.35	(34.86–51.53)

**Fig 3:**
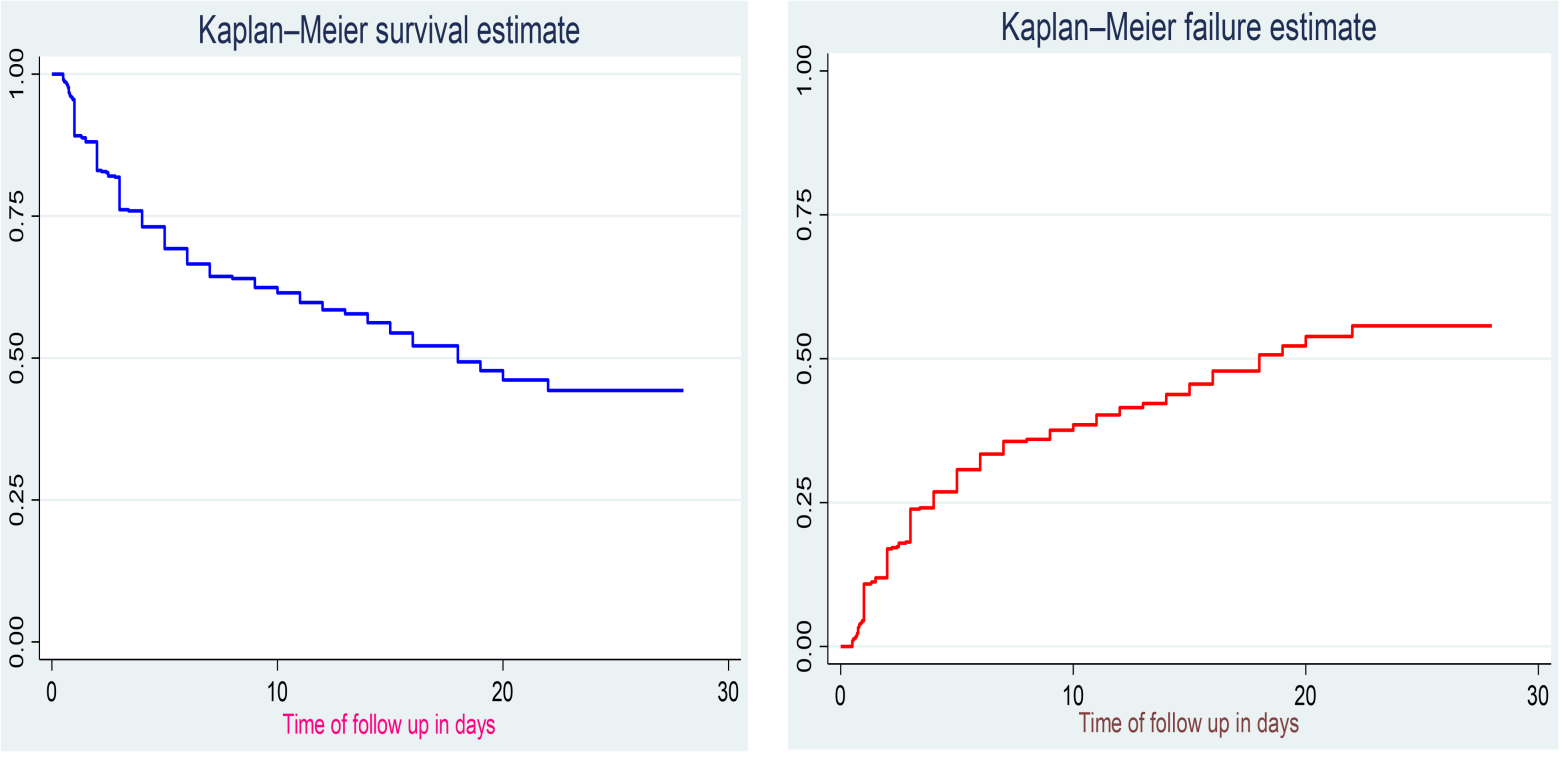
Overall Kaplan-Meier survival and failure estimates among preterm neonates admitted to the NICU of public hospitals in the Harari region and the Dire Dawa administration from November 1, 2021 to October 31, 2024.

The Kaplan-Meier curve illustrates the probabilities of survival and failure during follow-up time, indicating that these probabilities changed over time (Fig 3).

**Fig 4:**
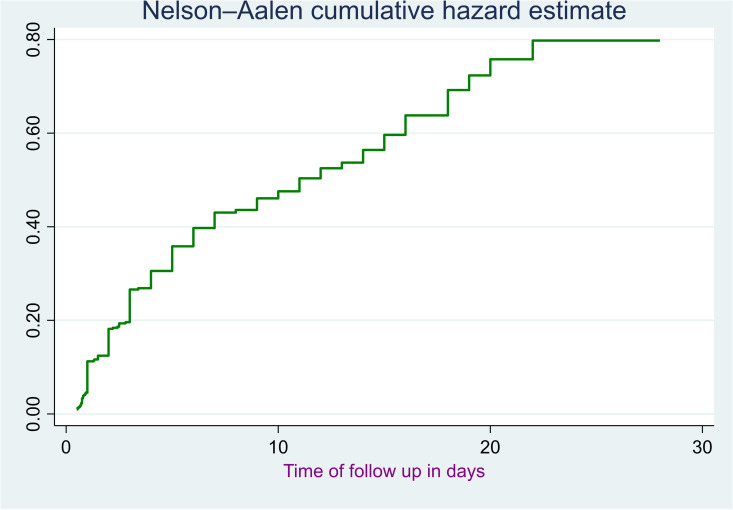
The overall Nelson-Aalen cumulative hazard estimate risk of mortality among preterm neonates admitted to the NICU of public hospitals in the Harari region and Dire Dawa administration from November 1, 2021 to October 31, 2024.

The Nelson-Aalen cumulative hazard estimate shows the accumulation of mortality risk over time among preterm neonates admitted to NICUs at public hospitals in the Harari region and Dire Dawa administration, up to 28 days of follow-up (Fig 4).

**Fig 5:**
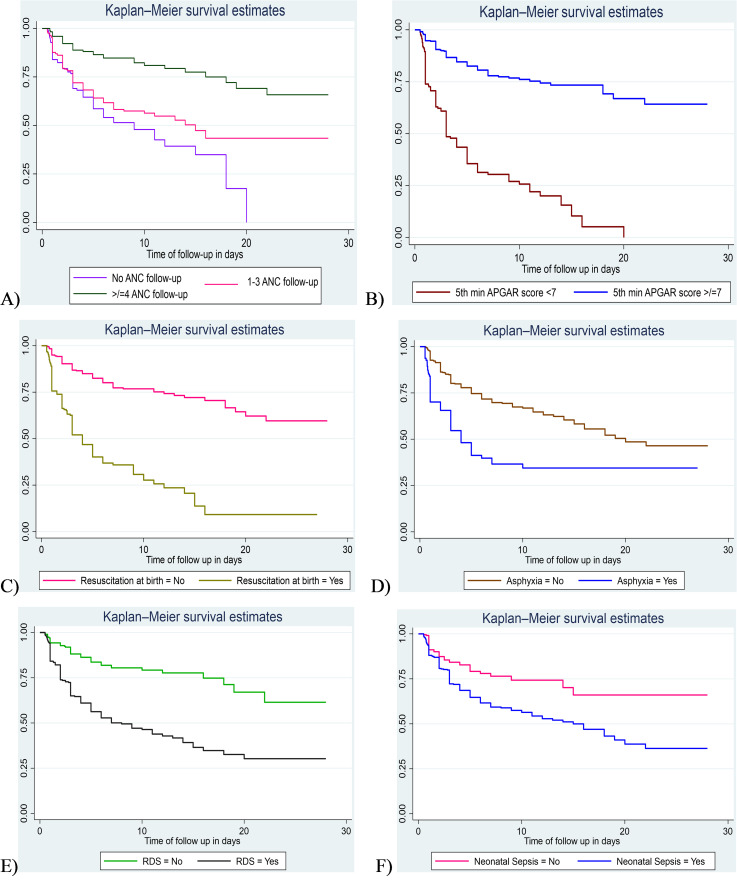
Kaplan-Meier survival curve compares the difference in survival time among exposed and unexposed (a, b, c, d, e, & f) preterm neonates admitted to the NICU of public hospitals in the Harari region and Dire Dawa administration from November 1, 2021, to October 31, 2024.

**Fig 6:**
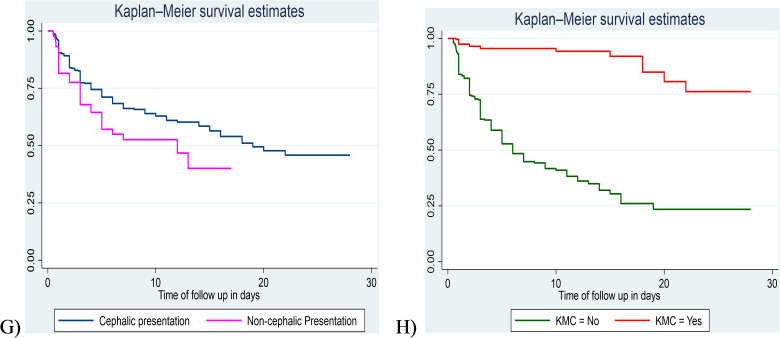
Kaplan-Meier survival curve compares the difference in survival time among exposed and unexposed (g & h) preterm neonates admitted to the NICU of public hospitals in the Harari region and Dire Dawa administration from November 1, 2021, to October 31, 2024.

The log-rank test was used for comparisons of survival time differences between different groups of categorical covariates. In this study, the log-rank test was statistically significant which shows the differences between groups in ANC follow up (Pr > X2 = 0.0000), 5th minute APGAR score (Pr > X2 = 0.0000), resuscitation with bag and mask at birth (Pr > X2 = 0.0000), PNA (Pr > X2 = 0.0000), RDS (Pr > X2 = 0.0000), neonatal sepsis (Pr > chi2 = 0.0001), presentation during birth (Pr > X2 = 0.0184) and using KM (Pr > X2 = 0.0000), as compared to their counterparts (Figs 5 and 6).

### Cox proportional hazards assumption test and model fitness test

**Fig 7:**
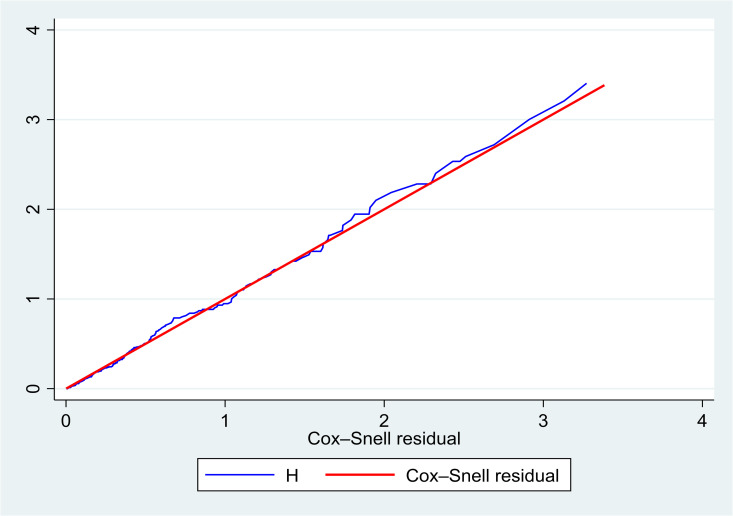
Cox-Snell residual for assessing the goodness-of-fit of the Cox proportional hazards model among preterm neonates admitted to the NICU of public hospitals in the Harari region and Dire Dawa administration from November 1, 2021 to October 31, 2024.

The proportional hazards assumption of the Cox model was evaluated using Schoenfeld residuals and a global test of proportionality. The global test was not statistically significant (Prob > χ² = 0.6401), indicating no evidence of violation of the proportional hazard assumption. Model adequacy was further assessed using Cox–Snell residuals, and the cumulative hazard plot closely followed the 45° reference line, suggesting good model fit (Fig 7).

### Predictor of preterm neonates’ mortality

As bivariable cox-regression at <5% significance level having of ANC contacts ≥ 4, grand multipara, having eclampsia, taking antenatal steroid, gestational age between 28 to < 32 and 32- < 34 weeks, < 1000gm weight of neonate, small weight for gestational age at birth, APGAR score at 1st and 5th minute < 7, diagnosed hypothermia with at admission, bag and mask resuscitation at birth, having perinatal asphyxia, having respiratory distress syndrome, having neonatal sepsis, non-cephalic presentation, neonates diagnosed with NEC, kangaroo mother care usage, and receiving CPAP were significantly predictors of mortality among preterm neonates compared to their counterparts. However, the multivariable Cox proportional hazards regression analysis identified having ≥ 4 ANC contacts, a fifth-minute APGAR score < 7, resuscitation with bag and mask at birth, perinatal asphyxia, respiratory distress syndrome, non-cephalic presentation, sepsis, and kangaroo mother care utilization as significant predictors of preterm neonatal mortality (Table 5).

**Table 5:** Multivariable Cox-Proportional Hazard Regression analysis among preterm neonates admitted to the NICU of public hospitals in the Harari region and Dire Dawa administration from November 1, 2021 to October 31, 2024 (n = 612).

Variables	Categories	Survival status		CHR 95% CI	AHR 95% CI
		Censored (n, %) n = 407	Death (n, %) n = 205		
Maternal age in years	15 – < 20	36 (70.59)	15 (29.41)	0.91 (0.54 - 1.55)	0.96(0.55–1.70)
	20 - 34	315(64.55)	173 (35.45)	1	1
	≥ 35	56 (76.71)	17 (23.29)	0.61 (0.37–1.00)	1.07(0.57 - 1.96)
ANC follow-up contacts	No ANC follow-up	73 (52.90)	65 (47.10)	1	1
	1-3 contacts	191 (63.88)	108 (36.12	0.77 (0.57–1.05)	1.04 (0.75–1.45)
	≥ 4contacts	143 (81.71)	32 (18.29)	0.28(0.18 - 0.42) ***	0.59 (0.36 - 0.96) *
Parity	Primipara	136 (64.15)	76 (35.85)	1	1
	Multipara	176(64.23)	98 (35.77)	0.93 (0.69 - 1.25)	0.84(0.59 - 1.19)
	Grand multipara	95 (75.40)	31 (24.80)	0.61(0.40 - 0.93) *	0.74 (0.43 - 1.29)
Bad obstetrics history	Yes	34 (54.84)	28 (45.16)	1.28(0.86 - 1.91)	1.02(0.65 - 1.58)
	No	373(67.82)	177 (32.18)	1	1
Chorioamnionitis	Yes	4(66.67)	2(33.33)	2.44(0.60–9.84)	1.09(0.25 - 4.89)
	No	403(66.50)	203 (33.50)	1	1
Preeclampsia	Yes	85(60.71)	55 (39.29)	1.31 (0.97 - 1.78)	1.04 (0.72 - 1.51)
	No	322(68.22)	150(31.78)	1	1
Eclampsia	Yes	21 (51.22)	20(48.78)	1.63 (1.03 - 2.59) *	1.08(0.64 - 1.82)
	No	386(67.60)	185 (32.40)	1	1
Steroid used	Yes	131(73.18)	48 (26.82)	0.66 (0.47 - 0.91) *	0.75(0.52 - 1.09)
	No	276(63.74)	157 (36.26)	1	1
Maternal anemia	Yes	39(63.93)	22 (36.07)	1.41 (0.91 - 2.20)	1.13(0.69 - 1.86)
	No	368(66.79)	183 (33.21)	1	1
Mothers with HIV	Reactive	4 (33.33)	8 (66.67)	2.02(0.99 - 4.02)	2.15(0.94 - 4.95)
	Non-reactive	403(67.17)	197 (32.83)	1	1
Gestational age in weeks	28 to < 32	62(42.18)	85 (57.82)	3.17(2.27 - 4.44) ***	1.42 (0.88 - 2.29)
	32 to <34	121(66.12)	62 (33.88)	1.63(1.14 - 2.33) **	1.23(0.81 - 1.86)
	34 to <37	224(79.43)	58 (20.57)	1	1
Weight of neonate (gm)	<1000	4(20)	16(80)	3.72 (1.46 - 9.55) **	1.25(0.38–4.08)
	1000-1499	76(44.71)	94 (55.29)	2.24 (0.98 - 5.12)	1.00(0.37 - 2.68)
	1500-2499	306(77.47)	89 (22.53)	0.84 (0.37 - 1.92)	0.68(0.28 - 1.66)
	≥2500	21 (77.78)	6(22.22)	1	1
Weight for gestational age at birth	Appropriate	312(72.22)	120 (27.78)	1	1
	Small	95 (52.78)	85 (47.22)	1.71(1.29 - 2.26) ***	0.72(0.49 - 1.06)
APGAR score at 1^st^ minute	<7	146(47.87)	159 (52.13)	4.60(3.31 - 6.40) ***	1.43(0.91 - 2.25)
	≥7	261 (85.02)	46 (14.98)	1	1
APGAR score at 5^th^ minute	<7	48(29.09)	117 (70.91)	5.28(3.98–7.00) ***	1.80(1.22 - 2.66) **
	≥7	359 (80.31)	88 (19.69)	1	1
Resuscitation at birth	Yes	68(36.76)	117 (63.24)	4.55(3.42–6.03) ***	1.59(1.10 - 2.29) *
	No	339(79.39)	88 (20.61)	1	1
PNA	Yes	41 (42.71)	55 (57.29)	2.48(1.82 - 3.38) ***	1.55(1.08 - 2.22) *
	No	366 (70.93)	150(29.07)	1	1
RDS	Yes	156 (50.49)	153 (49.51)	3.16(2.31 - 4.33) ***	1.75(1.22 - 2.51) **
	No	251(82.84)	52 (17.16)	1	1
Hypothermia diagnosed at admission	Yes	301(64.32)	167 (35.68)	1.53 (1.07–2.17) *	0.98 (0.67 - 1.44)
	No	106(73.61)	38 (26.39)	1	1
Hypoglycemia diagnosed at admission	Yes	38 (57.58)	28 (42.42)	1.30 (0.87 - 1.93)	1.27(0.81–2.01)
	No	369(67.58)	177 (32.42)	1	1
Jaundice	Yes	75(66.96)	37 (33.04)	0.77 (0.54 - 1.10)	0.86(0.58 - 1.28)
	No	332(66.40)	168 (33.60)	1	1
Neonatal sepsis	Yes	227(58.21)	163 (41.79)	1.9 (1.36 - 2.69) ***	1.58 (1.09 - 2.28) *
	No	180(81.08)	42(18.92)	1	1
Type of presentation	Cephalic	353(67.50)	170(32.50)	1	1
	Non-cephalic	54(60.67)	35(39.33)	1.53 (1.06 - 2.21) *	1.68(1.12 - 2.53) *
Neonates diagnosed with NEC	Yes	16(33.33)	32(66.67)	2.10(1.42 - 3.02) ***	1.24(0.80 - 1.92)
	No	391(69.33)	173(30.67)	1	1
Kangaroo mother care usage	Yes	220(93.22)	16 (6.78)	0.09(0.05 - 0.15) ***	0.16(0.09 −0.27) ***
	No	187(49.73)	189(50.27)	1	1
CPAP	Yes	212(60.23)	140(39.77)	1.58 (1.18 - 2.12) **	0.77 (0.55 - 1.08)
	No	195(75)	65(25)	1	1

**Note:** AHR = Adjusted Hazard Ratio, CHR = Crude Hazard Ratio, CI = Confidence Interval, 1 = Reference group, p-value < 0.05 (*), p-value < 0.01 (**), and p-value < 0.001 (***).

Findings from the multivariable Cox proportional hazards regression analysis revealed that the hazard of death among preterm neonates born to mothers with ≥ 4 ANC contacts was 41% lower compared to those born to mothers who had no ANC contacts (AHR = 0.59; 95% CI: 0.36–0.96). The hazards of death among preterm neonates with APGAR score at 5th minute <7 were 1.80 times higher than those with APGAR score at 5th minute ≥7 (AHR = 1.80; 95% CI: (1.22–2.66). Preterm neonates who were resuscitated with bag and mask at birth had 1.59 times higher risk of mortality compared to those who were not resuscitated (AHR = 1.59; 95% CI: 1.10–2.29). The risk of death of preterm neonates with perinatal asphyxia (PNA) was 1.55 times higher than that of those who had no PNA (AHR = 1.55; 95% CI: 1.08–2.22).

Similarly, preterm neonates diagnosed with respiratory distress syndrome (RDS) had a 75% higher risk of mortality compared to those without RDS (AHR = 1.75; 95% CI: 1.22–2.51). Preterm neonates with non-cephalic presentation at birth had 1.68 times higher risk of death compared to those with cephalic presentation (AHR = 1.68; 95% CI: 1.12–2.53). The hazard of death of preterm neonates with neonatal sepsis was 1.58 times higher than that of those without sepsis (AHR = 1.58; 95% CI: 1.09–2.28). Likewise, preterm neonates who received KMC had an 84% lower risk of death compared to those who did not receive KMC (AHR = 0.16; 95% CI: (0.09–0.27) (Table 5).

## Discussion

This study found that the cumulative incidence of preterm neonatal mortality rate at the end of the follow-up period was 33.50% (95% CI: 29.76–37.39), with an incidence rate of 52.76 (95% CI: 46.01–60.50) deaths per 1000 preterm neonate-days. The median survival time for preterm neonates was 18 days.

The incidence of preterm neonatal mortality observed in this study was consistent with findings from various hospitals across Ethiopia and other regions. Similar results were documented in studies conducted in Addis Ababa [31], Bench Sheko Zone, Sheka Zone and Keffa Zone [30], and Mizan-Tepi University Teaching Hospital [28], Tigray region hospital [32], Felege Hiwot Hospital in Bahir Dar city [33], and Fort Portal Regional Referral Hospital in Uganda [11].

However, this finding was higher than the study conducted in those upper-middle- and high-income countries, including in China [34,35], Iran [36] and Saudi Arabia [37]. The distinction could be due to economic and sociodemographic differences between Ethiopia and those nations. Infants born preterm in those countries have received improved care during the pre-pregnancy, antenatal, intrapartum, and postnatal periods [38]. These economic and sociodemographic differences may lead to differences in the quality-of-service provision in preterm neonatal care, and developed countries might be better equipped with skilled professionals, a support labor force, and advanced equipment necessary for preterm neonatal care [24,27].

The findings of this study revealed a higher incidence of preterm neonatal mortality compared to studies conducted in Uganda [39], western Sierra Leone [40], Nigeria [41], Jordan [42], and a multicenter study in India and Pakistan [43]. Similarly, within Ethiopia, lower preterm mortality rates were reported from Aksum Referral and General Specialty Hospital [44], a multicenter study conducted in five hospitals [16], Jimma University Medical Center [45], public hospitals in the west Guji and Borena zones [27], the University of Gondar Comprehensive Specialized Hospital [25], and Tikur Anbesa Specialized Hospital [24]. The variation in findings compared to studies from Jordan and a multicenter study in India and Pakistan may be due to differences in economic status, healthcare facilities, study settings, and quality of services [45]. Differences within studies from Ethiopia, Nigeria, Sierra Leone, and Uganda could also reflect variations in service quality among hospitals, with some offering more specialized care [28,30]. Additionally, the larger sample size in the current study may have contributed to the higher reported mortality rate.

In this study, the overall incidence rate of preterm neonatal death was 52.76 (95% CI: 46.01–60.50) per 1,000 preterm neonate-days, with a median survival time of 18 days. This finding is comparable to a previous study conducted in public hospitals in the West Guji and Borena zones, which also reported a median survival time of 18 days [27]. This may be due to similarities in study populations, designs, and healthcare settings across the regions.

The incidence rate of preterm neonatal mortality in this study was higher than that reported in the Busoga region of Uganda [39], two specialized hospitals in the Tigray region [32], Tikur Anbesa Specialized Hospital, with a median survival time of 21 days [24], Nigist Eleni Mohammed Memorial Hospital [46], and Jimma University Medical Center [45]. This variation could be explained by differences in health care quality, patient demographics, referral patterns, or availability of neonatal intensive care services across the various study sites.

Conversely, the incidence rate observed in this study was lower than that reported in studies conducted in Bench Sheko, Sheka, and Keffa Zones [30] and at Mizan Tepi University Teaching Hospital [28]. The lower discrepancy may be explained by variations in study setting, design, quality of care, and healthcare access among those hospitals and the current study hospitals.

This study also identified predictors of mortality among preterm neonates. Accordingly, preterm neonates born to mothers who had four or more ANC contacts had a 44% lower risk of death compared to those whose mothers had fewer than four ANC contacts (AHR = 0.56; 95% CI: 0.36–0.89). This finding is supported by studies conducted at the University Teaching Hospital of Butare in Rwanda [47] and in South Western Uganda [48]. This might be because of the protective effect of adequate antenatal care, likely due to improved maternal health monitoring, early detection of complications, and timely interventions [49]. Additionally, regular ANC visits enhance maternal awareness of danger signs and promote maternal and fetal health in reducing antenatal pregnancy complications, which were associated with preterm neonatal death [50].

In this study, preterm neonates who had a fifth-minute APGAR score of <7 were 1.80 times more likely at hazard of death than those who had an APGAR score of ≥7 (AHR = 1.80; 95% CI: 1.22–2.66). This finding is supported by studies conducted in tertiary hospitals in Ghana [51] and Sierra Leone [40], as well as in multiple hospitals in China [34] and Iran [36,52]. Similarly, studies from Ethiopian facilities, including Mizan Tepi University Teaching Hospital [28] and Tikur Anbessa Specialized Hospital in Addis Ababa aligns with the current finding [24]. This may be attributed to the fact that the 5-minute APGAR score assesses how well the newborn is adapting to the extrauterine environment. A low score at this stage suggests poor physiological adaptation, which increases the risk of neonatal mortality [53]. Moreover, neonates with a fifth-minute APGAR score below 7 often require intensive and specialized care. In settings where timely and advanced medical support from skilled professionals equipped with appropriate resources is lacking, these newborns are at a substantially increased risk of mortality compared to those with higher APGAR scores [28,51].

From the current study, preterm neonates who had perinatal asphyxia (PNA) were 1.55 times more at risk of mortality compared to their counterparts (AHR = 1.55; 95% CI: 1.08–2.22). This finding is consistent with previous studies conducted in China [7,34], at Mbarara Regional Referral Hospital in Southwestern Uganda [48] and in several Ethiopian institutions, including the University of Gondar Comprehensive Specialized Hospital [25], and comprehensive specialized hospitals in the Tigray region [32]. The possible explanation was that perinatal asphyxia remains a major cause of neonatal death in the country due to poor-quality and limited emergency obstetric and newborn care services. Perinatal asphyxia can also damage vital organs, disrupt gas exchange, and worsen organ immaturity in preterm neonates, increasing the risk of death. Additionally, it often leads to severe complications like hypoxic-ischemic encephalopathy and intraventricular hemorrhage, especially in preterm infants [16,53].

In the current study, preterm neonates who received bag-and-mask resuscitation at birth had a 59% higher hazard of death compared to those who were not resuscitated (AHR = 1.59; 95% CI: 1.10–2.29). This finding is supported by evidence from studies conducted in public hospitals in southern Ethiopia [27], Kenya [54], and western Uganda [11]. The possible reason is that the need for resuscitation often indicates serious underlying conditions linked to poor neonatal outcomes, and in low-resource settings, inadequate resuscitation quality and limited post-care contribute to higher mortality in resuscitated preterm neonates. Moreover, resuscitation may act as a pathway for microbial entry, leading to infection, as preterm neonates generally have weak immune defenses, which can lead to neonatal sepsis, which is significantly associated with preterm neonatal mortality [11,55,56].

In this study, preterm neonates diagnosed with RDS had a 1.75 times higher hazard of mortality compared to those without RDS (AHR = 1.75; 95% CI: 1.22–2.51). This finding is supported by studies conducted in southwestern and western Uganda [11,48] and is consistent with research from various parts of Ethiopia, including Addis Ababa [24], Bahir Dar [33], Gondar [25], and Mizan Tepi [28]. The reason is that the increased risk of mortality among preterm neonates with RDS can be attributed to the immaturity of the lungs, particularly due to insufficient production of pulmonary surfactant, a crucial substance that reduces surface tension and prevents alveolar collapse at the end of expiration [53,57].

The hazard of death among preterm neonates born in a non-cephalic presentation was 1.7 times higher than that of those born in cephalic presentation (AHR = 1.68; 95% CI: 1.12–2.53). This finding is supported by studies conducted in various countries and settings, with similar results reported in Uganda [48], in Iran [58], Mizan-Tepi University Teaching Hospital in Ethiopia [28], and a multicenter study in Ethiopian public hospitals [59]. This could be attributed to the increased risk of trauma during delivery, asphyxia, and meconium aspiration syndrome among preterm neonates delivered in a non-cephalic presentation at an early gestational age, which may subsequently result in complications and death in preterm neonates [16,28].

Furthermore, in this study, preterm neonates with neonatal sepsis had a 58% higher risk of mortality compared to those without sepsis (AHR = 1.58; 95% CI: 1.09–2.28). This finding is supported by studies conducted at Tikur Anbessa Specialized Hospital in Addis Ababa [24] and Jimma University Medical Center [45]. This may be attributed to the increased susceptibility of preterm neonates to infections, as their immature immune systems, coupled with inadequate calorie intake, make them more vulnerable and potentially raise the risk of death [60].

Moreover, in the current study, preterm neonates who received kangaroo mother care had an 84% lower hazard of mortality compared to those who did not receive it (AHR = 0.16; 95% CI: 0.09–0.27). This finding is consistent with studies conducted in the southwestern and Busoga regions of Uganda [39,48], Jimma [45], Southwest Ethiopia [30], Addis Ababa [31], Gondar [25], and the Tigray region [32]. This might be because most preterm neonates in those studies did not receive KMC. KMC plays a vital role in protecting neonates from infections, effectively managing hypothermia, enhancing gastrointestinal function and cardiorespiratory stability, and promoting the initiation and continuation of breastfeeding [61].

## Strengths and limitations

This study showed survival status and indicated important predictors of mortality among preterm neonates, contributing to the evidence for early identification and management of preterm neonates with identified complications. Since this study involved time-to-event analysis, it allowed for the inclusion of censored study subjects in assessing their contributions. It was conducted in two major cities in eastern Ethiopia with a relatively large sample size. The limitation of this study is follow-up is only limited until discharge. Some events may occur after discharge. Due to the incomplete records, certain major predictors of preterm mortality, such as all maternal demographics, nutritional conditions, and institutional factors, were not addressed.

## Conclusion

The study found a high incidence of preterm neonatal mortality, with most deaths occurring in the early neonatal period. The main contributing conditions were hypothermia, neonatal sepsis, and respiratory distress syndrome (RDS). Key predictors of mortality included fewer than four ANC visits, lack of kangaroo mother care (KMC), non-cephalic presentation, neonatal sepsis, RDS, perinatal asphyxia, low APGAR score, and need for bag-and-mask resuscitation at birth.

Most predictors are preventable with available facility resources. Strengthening ANC attendance, promoting KMC, and improving integrated neonatal care, particularly resuscitation, infection prevention, and management of asphyxia, RDS, and sepsis, are essential. Further research should investigate maternal, socioeconomic, nutritional, and institutional factors affecting preterm neonatal survival.

## Abbreviations

ANC: Antenatal Care
AHR: Adjusted Hazard Ratio
APGAR: Appearance, Pulse, Grimace, Activity, Respiration
CPAP: Continuous Positive Airway Pressure
KMC: Kangaroo Mother Care
NICU: Neonatal Intensive Care Unit
PNA: Perinatal Asphyxia
RDS: Respiratory Distress Syndrome
WHO: World Health Organization
